# Glycopenia - induced sympathoadrenal activation in diabetes mellitus and uncontrolled arterial hypertension: an observational study

**DOI:** 10.1186/s13098-020-00613-4

**Published:** 2020-11-30

**Authors:** Abimbola Abobarin-Adeagbo, Andreas Wienke, Matthias Girndt, Rainer U. Pliquett

**Affiliations:** 1 Department of Internal Medicine II, Martin-Luther-Universität Halle-Wittenberg, Universitätsklinikum Halle, Ernst-Grube-Str. 40, 06120 Halle (Saale), Germany; 2 Institute of Medical Epidemiology, Biometry and Informatics, Martin-Luther-University Halle-Wittenberg, Halle (Saale), Germany; 3 Department of Nephrology & Diabetology, Carl-Thiem Hospital, Cottbus, Germany

**Keywords:** Arterial hypertension, Norepinephrine, Hypoglycemia, Diabetes mellitus

## Abstract

**Background:**

Aim of this study is to investigate a possible association of hypoglycemic episodes and arterial hypertension. We hypothesize that hospitalized insulin-treated diabetes patients with hypertensive crisis have more hypoglycemic episodes than their counterparts without hypertensive crisis on admission.

**Methods:**

In a prospective, observational cohort study, 65 insulin-treated diabetes patients (type 1, type 2, type 3c) were included in Group 1, when a hypertensive crisis was present, as control patients in Group 2 without hypertensive crisis or hypoglycemia, in Group 3, when a symptomatic hypoglycemia was present on admission. All patients were subjected to open-label continuous glucose monitoring, 24-h blood-pressure- and Holter electrocardiogram recordings, and to laboratory tests including plasma catecholamines.

**Results:**

53 patients, thereof 19 Group-1, 19 Group-2, 15 Group-3 patients, completed this study. Group-1 patients had the highest maximum systolic blood pressure, a higher daily cumulative insulin dose at admission, a higher body-mass index, and a higher plasma norepinephrine than control patients of Group 2. Group-3 patients had more documented hypoglycemic episodes (0.8 ± 0.5 per 24 h) than Group-2 patients (0.2 ± 0.3 per 24 h), however, they were not different to the ones in Group-1 patients (0.4 ± 0.4 per 24 h). Plasma norepinephrine and mean arterial blood pressure were higher Group-1 and Group-3 patients than in control patients of Group 2. At discharge, the daily cumulative insulin dose was reduced in Group-1 (− 18.4 ± 24.9 units) and in Group-3 patients (− 18.6 ± 22.7 units), but remained unchanged in Group-2 control patients (− 2.9 ± 15.6 units).

**Conclusions:**

An association between hypoglycemic events and uncontrolled hypertension was found in this study.

## Background

Randomized clinical trials have proven that the use of intensive insulin therapy to target normal glycated hemoglobin A1c (HbA1c) did not offer cardiovascular benefits [1] and have been shown to even increase the cardiovascular mortality for type-2-diabetes patients [2]. The reason for this lack of benefit or even excess mortality seen, may relate to hypoglycemia, which correlates with the occurrence of ventricular arrhythmias [3] and bradycardia [4]. Although hypoglycemic events occurred less frequently in type-2- than in type-1 diabetics, the related mortality risk was higher for type-2 diabetics [5].

With the advent of skin-based CGM, the more inclusive term “glycopenia” reflects a shortage of glucose in tissues such as the skin or the brain. In fact, after a certain time delay when reaching a steady state, the terms glycopenia and hypoglycemia can be used interchangeably, as blood glucose and tissue glucose level off in the same range. Blood glucose or tissue glucose below 70 mg/dL (3.9 mmol/L) is regarded as hypoglycemia or glycopenia, as it has been recognized as a threshold for neuroendocrine responses to falling glucose in people without diabetes [6]. Sequelae of hypoglycemia include a neurohormonal stimulation leading to a post-hypoglycemic hyperglycemia, a term previously coined as “Somogyi effect” [7]. Specifically, neuroglycopenia translates into a neurohormonal activation involving both the hypothalamic-pituitary axis and the sympathetic nervous system [8]. An association between hypoglycemia and arterial hypertension has been demonstrated in a small cohort study of type-1- and type-2-diabetes patients on a continuous glucose monitoring, while concurrently performing a 24-h ambulatory blood pressure monitoring (ABPM) [9]. Given the correlation between hypoglycemia and arterial hypertension, we hypothesized that consecutively hospitalized insulin-treated diabetes patients with hypertensive crisis on admission have a propensity for hypoglycemic episodes in post-admission continuous glucose monitoring (CGM). As a secondary hypothesis, plasma norepinephrine concentrations are expected to be elevated both in insulin-treated diabetes patients with hypertensive crisis and in diabetes patients with hypoglycemia on admission. To test these hypotheses, we recruited hospitalized insulin-treated diabetes patients presenting with hypertensive crisis at admission. As negative-control group, insulin-treated diabetics with neither hypoglycemia nor hypertensive crisis at admission were included. Diabetes patients, who presented with symptomatic hypoglycemia, served as the positive-control group. Overall, the results of this study may highlight a possible association between uncontrolled arterial hypertension and a propensity of hypoglycemic events.

## Methods

65 Insulin-treated diabetes patients (type 1, type 2, type 3c), hospitalized in the University Hospital Halle (Saale) between 1.6.2017 and 31.12.2019 were screened and enrolled for participation in this observational cohort study based on the inclusion and exclusion criteria. The number of recruited study participants was limited by in- and exclusion criteria. Due to the scheduled time frame of study, a power calculation for study size was not possible. All patients provided written informed consent and staff physicians treating the patients did not participate in this study in any way. Decisions were not influenced by the organizers or by persons conducting this observational study. The ethics committee of the Medical Faculty of the Martin-Luther University Halle-Wittenberg approved this study protocol (Study number 2017–28). Data acquisition was performed according to the principles of the Declaration of Helsinki and Good Clinical Practice (E6, revision 2) from 2015.

### Inclusion criteria

- Insulin-treated diabetes patients (type 1, type 2 or Type 3c Diabetes mellitus), diagnosed for at least one year before study enrollment,
- Age: 18–99 years
- Male or female

Cohort specific inclusion criteria:

- Group 1: hypertensive crisis at admission (systolic blood pressure > 180 mmHg)
- Group 2: absence of hypertensive crisis or symptomatic hypoglycemia at admission.
- Group 3: symptomatic hypoglycemia at admission

### Exclusion criteria

- Age below 18 years or more than 99 years.
- Tumor disease or curative care within 5 years,
- Pregnancy or women with child-bearing potential with no safe forms of contraception,
- Pain (visual analogue scale from 1–10: > 3),
- Known secondary cause of arterial hypertension,
- Septicaemia,
- Allergies to adhesives, inability to use CGM.
- Use of glucocorticoids,
- Psychiatric disorders and all forms of dementia with lack of ability to provide an informed consent, -chronic kidney disease (defined by estimated glomerular filtration rate (eGFR) < 15 ml/min, stage G5 according to Kidney Disease: Improving Global Outcomes [10]).
- Acute kidney injury (AKI) necessitating renal-replacement therapy.
- Acute or chronic heart failure (New York Heart Association class higher than 2).

### Study visits

#### Visit 1

Within 24 h after admission, f informed consent taking, study recruitment and group allocation were done. Medical history taking and clinical examination were carried out. Patients received instructional materials and behavioral counseling regarding diabetes care. In addition, a CGM sensor (FreeStyle libre, Abbott Diabetes Care, Abbott GmbH, Wiesbaden, Germany), a 24-h ABPM and Holter electrocardiogram were installed.

#### Visit 2

Within 48 h after admission, venous blood glucose, plasma catecholamines, serum cortisol, and routine laboratory parameters including serum creatinine and HbA1c were determined.

#### Visit 3

Prior to discharge or 14 days after Visit 2 (whatever applied first), the CGM sensor was removed, data was retrieved and analyzed. Concomitant medications including daily cumulative insulin dose and laboratory parameters including serum creatinine, eGFR, if applicable, were recorded. In case of an evolving AKI as shown by an increase (> 0.3 mg/dL) of serum creatinine by discharge, eGFR was not calculated. In case of an AKI prior to hospitalization as shown by a decrease (> 0.3 mg/dL) of serum creatinine by discharge or in case of no change (> 0.3 mg/dL) of serum creatinine by discharge, eGFR at discharge was provided.

### Analysis

If applicable, both the initial hypertensive crisis and the symptomatic hypoglycemia at admission were considered for cohort allocation only, not for analysis. Information on missing data was provided in the Tables. Continuous data were given as mean ± standard deviation. To test for normality, Kolmogorov–Smirnov test was used. For group-wise comparisons, an ordinary one-way Analysis of Variance test was used as a parametric test, Kruskal–Wallis test was used, if data showed no Gaussian distribution. As post-hoc tests, Tukey or Dunn`s test were used, where appropriate.

### Primary outcome parameters

Number of post-admission hypoglycemic episodes (tissue glucose level < 3.9 mmol/L) per 24 h of CGM. Symptomatic hypoglycemic episodes were confirmed by blood-glucose tests. The duration of hypoglycemic episodes was not considered for analysis.

### Secondary outcome parameters

- Change in classes and defined daily dose (DDD) of antihypertensive medications, by discharge,
- Change in daily cumulative insulin dose by discharge
- Comparison of plasma catecholamines as a surrrogate of sympathetic tone, heart rate variability (the standard deviation of RR intervals derived from Holter electrocardiogram) as a surrogate of parasympathetic tone [11]
- HbA1c among groups
- Body-mass index among groups
- eGFR among groups

## Results

**Fig. 1:**
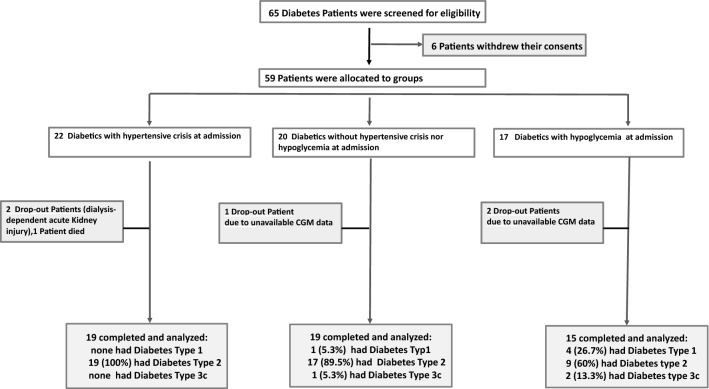
Flow chart which demonstrates screening and study recruitment of diabetes patients to this study

**Fig. 2:**
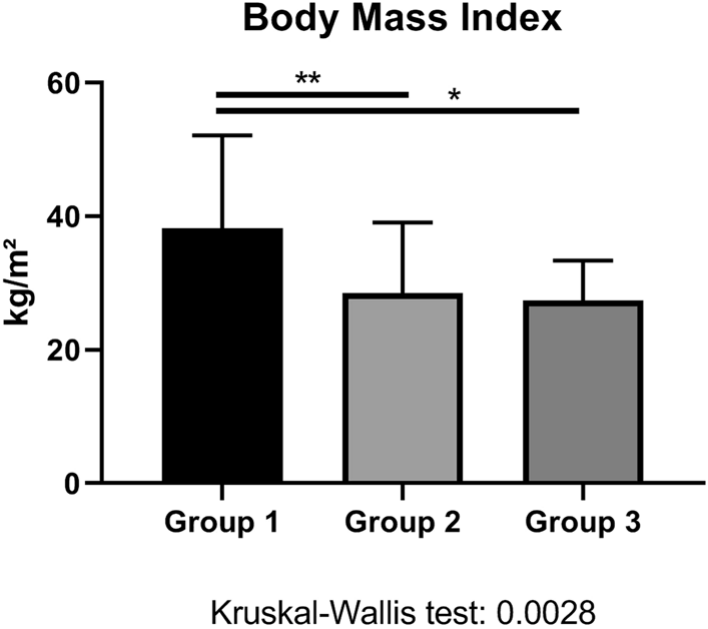
Body–Mass index among groups. Asterisks represent p values in post-hoc analysis

**Fig. 3:**
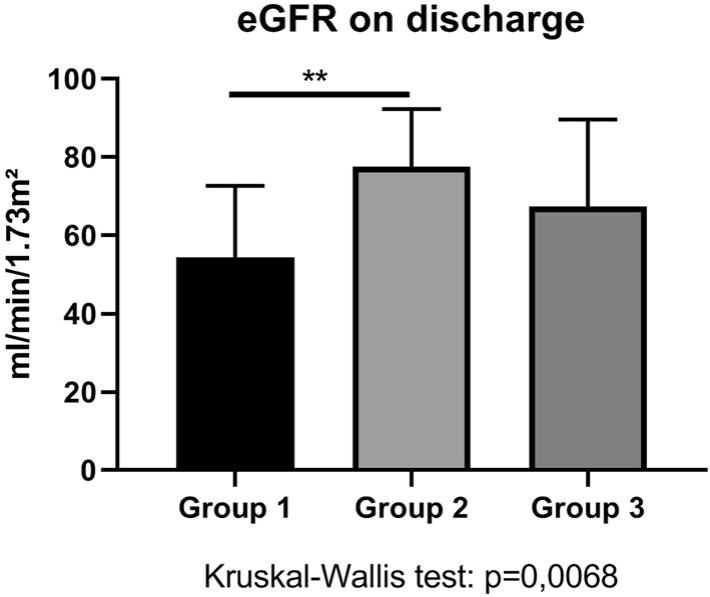
Estimated glomerular filtration function among groups. Asterisks represent p values in post-hoc analysis

53 diabetes patients on insulin therapy completed this prospective observational study (Fig. 1). The key baseline characteristics are represented in Table 1. Group-1 patients were more obese than Group-2 or Group-3 patients (Fig. 2), and they had an impaired renal function when compared to the negative control Group 2 (Fig. 3). The concomitant antihypertensive medication did not differ between groups at admission (Table 1). However, the average daily cumulative insulin dose was higher in Group-1 patients than in the control patients Group 2 (Table 2).

**Table 1:** Baseline characteristics of hospitalized diabetes patients with a hypertensive crisis (Group 1), without a hypertensive crisis or a symptomatic hypoglycemia (Group 2), with symptomatic hypoglycemia (Group 3) at admission

	Group 1			Group 2			Group 3			
	n	Mean ± SD	n^a^	n	Mean ± SD	n^a^	n	Mean ± SD	n^a^	p
Men/women (n)	7/12	NA	NA	9/10	NA	NA	6/9	NA	NA	NA
Diabetes type 1/2/3c	0/19/0	NA	19	1/17/1	NA	19	6/8/1	NA	15	NA
Age (years)	19	69.9 ± 9.8	19	19	64.1 ± 15.8	19	15	62.2 ± 21.9	15	0.3449
Body mass index (kg/m^2^)	19	38.1 ± 14.0	19	19	28.5 ± 10.5	19	15	27.4 ± 6.0	15	0.0028
Antihypertensive classes per patient at admission (n)	19	3.9 ± 1.6	19	19	2.3 ± 1.6	19	15	2.5 ± 1.6	15	0.1435
Antihypertensive DDD at admission (n)	19	6.2 ± 5.4	19	19	3.0 ± 3.4	19	15	4.7 ± 6.0	15	0.0662
Daily cumulative insulin dose (units/d)	19	60.9 ± 41.5	19	19	30.5 ± 27.6	19	15	46.5 ± 23.3	15	0.0229
HbA1c (%)	19	8.6 ± 2.8	19	19	8.9 ± 2.8	19	15	7.7 ± 1.5	15	0.8592
Urea (plasma; mmol/L)	19	10.5 ± 5.1	19	19	6.6 ± 5.0	19	15	6.9 ± 4.2	15	0.0105
Creatinine (serum; µmol/L)	19	132.8 ± 55.3	19	19	89.5 ± 37.2	19	15	110.5 ± 55.2	15	0.0106
Cortisol (plasma; pg/mL)	19	395.4 ± 156.9	17	19	331.1 ± 126.7	18	15	387.6 ± 176.1	13	0.4079
Epinephrine (plasma; pg/mL)	19	34.9 ± 26.1	18	19	32.6 ± 20.4	18	15	32.6 ± 24.4	14	0.9326
Norepinephrine (plasma; pg/mL)	19	788.6 ± 411.9	17	19	437.5 ± 239.3	17	15	644.3 ± 378.7	14	0.0191
Maximal heart rate (bpm)	19	107.6 ± 18.8	13	19	102.2 ± 23.3	11	15	119.1 ± 24.5	9	0.2373
Mean heart rate (bpm)	19	79.2 ± 13.4	13	19	72.8 ± 15.8	11	15	82.8 ± 19.6	9	0.3751
Minimal heart rate (bpm)	19	66.7 ± 13.2	13	19	58.8 ± 13.3	11	15	63.0 ± 14.8	9	0.3844
Heart rate variability, standard deviation of heart-beat intervals (ms)	19	65.1 ± 52.5	14	19	60.5 ± 40.4	15	15	56.4 ± 35.5	9	0.9584

^a^Final number of diabetes patients subjected to statistical analysis, if data were lacking

**Table 2:** Outcomes of hospitalized diabetes patients with a hypertensive crisis (Group 1), without a hypertensive crisis or a symptomatic hypoglycemia (Group 2), with symptomatic hypoglycemia at admission (Group 3)

	Group 1			Group 2			Group 3			
	n	Mean ± SD	n^a^	n	Mean ± SD	n^a^	n	Mean ± SD	n^a^	p
Renal function										
Creatinine at discharge (serum; µmol/L)	19	114.5 ± 36.6	17	19	89.8 ± 32.6	19	15	100.2 ± 46.5	13	0.0754
Change of serum creatinine (baseline versus discharge; µmol/l)	19	-17.9 ± 41.6	17	19	0.3 ± 44.1	19	15	-12.4 ± 65.4	13	0.6705
Estimated glomerular filtration rate (ml/min/1.73m^2^) at discharge	19	55.2 ± 18.6	16	19	76.7 ± 14.9	16	15	67.3 ± 22.4	12	0.0068
Blood pressure during hospitalization										
Systolic blood pressure (maximum, day-time; mmHg)	19	191.6 ± 20.7	19	19	157.7 ± 20.2	18	15	172.8 ± 22.2	15	< 0.0001
Systolic blood pressure (mean, day-time; mmHg)	19	142.5 ± 13.8	19	19	124.6 ± 16.1	19	15	138.7 ± 18.2	15	0.0030
Diastolic blood pressure (maximum, day-time; mmHg)	19	98.7 ± 13.4	19	19	88.6 ± 11.4	18	15	98.9 ± 20.0	15	0.0749
Diastolic blood pressure (mean, day-time; mmHg)	19	75.3 ± 9.4	19	19	71.3 ± 8.3	19	15	79.1 ± 15.4	15	0.1340
Mean arterial pressure (day-time; mmHg)	19	97.7 ± 8.9	19	19	85.9 ± 9.5	16	15	98.9 ± 15.3	15	0.0035
Systolic blood pressure (maximum, night-time; mmHg)	19	167.7 ± 30.4	19	19	135.6 ± 15.1	15	15	151.5 ± 23.2	15	0.0021
Systolic blood pressure (mean, night-time; mmHg)	19	141.7 ± 18.6	19	19	117.9 ± 12.1	16	15	125.1 ± 17.8	15	0.0003
Diastolic blood pressure (maximum, night-time; mmHg)	19	90.9 ± 15.7	19	19	81.3 ± 8.2	16	15	90.8 ± 15.7	15	0.1339
Diastolic blood pressure (mean, night-time; mmHg)	19	72.9 ± 11.3	19	19	68.7 ± 8.6	16	15	71.7 ± 12.9	15	0.5279
Mean arterial pressure (night-time; mmHg)	19	95.9 ± 11.9	19	19	85.9 ± 9.5	16	15	89.5 ± 13.8	15	0.0507
Antihypertensive classes per patient at discharge (n)	19	4.1 ± 1.6	19	19	2.2 ± 1.6	19	15	2.2 ± 1.2	15	0.0011
Change of antihypertensive classes (baseline versus discharge; n)	19	1.1 ± 1.1	19	19	0.2 ± 1.1	19	15	-0.3 ± 1.0	15	0.0017
Antihypertensive DDD at discharge (n)	19	11.0 ± 8.7	19	19	3.0 ± 3.3	19	15	4.9 ± 6.5	15	0.0012
Change of antihypertensive DDD (baseline versus discharge; n)	19	4.8 ± 6.1	19	19	0.1 ± 1.9	19	15	0.2 ± 3.8	15	0.0028
Diabetes-related parameters										
Length of CGM (d)	19	5.1 ± 0.7	19	19	5.3 ± 1.5	19	15	6.2 ± 0.9	15	0.0004
Hypoglycemic episodes^a^ (during CGM, n)	19	2.2 ± 1.9	19	19	0.7 ± 1.4	19	15	4.5 ± 2.3	15	< 0.0001
Hypoglycemic episodes per night^a^ (during CGM, n)	19	0.8 ± 1.0	19	19	0.2 ± 0.5	19	15	1.5 ± 1.4	15	0.0051
Daily cumulative insulin dose at discharge (units/d)	19	42.5 ± 33.0	19	19	27.6 ± 24.3	19	15	27.9 ± 19.0	15	0.2960
Change of daily cumulative insulin dose (baseline versus discharge; units/d)	19	-18.4 ± 24.9	19	19	-2.9 ± 15.6	17	15	-18.6 ± 22.7	15	0.0479
Primary outcome										
Hypoglycemic episodes (tissue glucose < 3.9 mmol/l) per 24 h (n)	19	0.4 ± 0.4	19	19	0.2 ± 0.3	17	15	0.8 ± 0.5	15	< 0.0001

^a^Final number of diabetes patients subjected to statistical analysis, if data were lacking

### Insulin-treated diabetes patients with hypertensive crisis (Group 1) and with symptomatic hypoglycemia (Group 3) were not different in terms of hypoglycemic burden during hospitalization

**Fig. 4:**
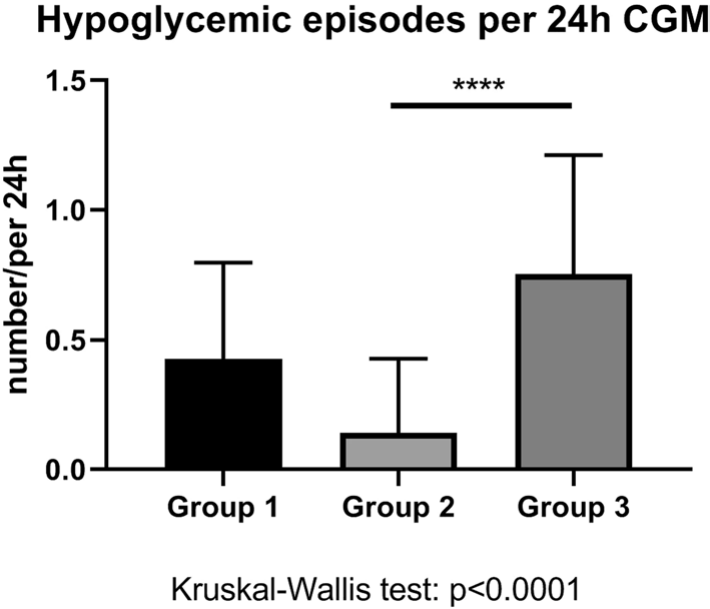
Hypoglycemic episodes per 24 h continuous glucose monitoring (CGM) among groups. Asterisks represent p values in post-hoc analysis

**Fig. 5:**
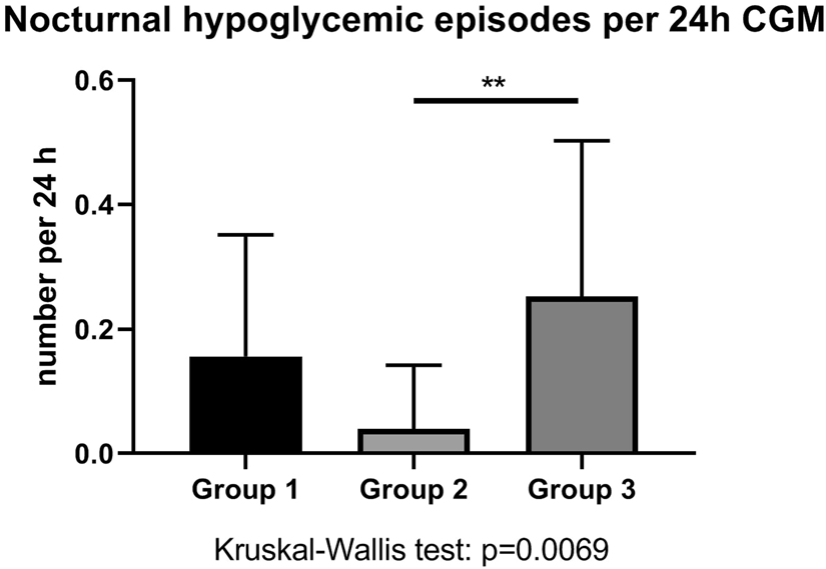
Nocturnal hypoglycemic episodes per 24 h continuous glucose monitoring (CGM) among groups. Asterisks represent values in post-hoc analysis

**Fig. 6:**
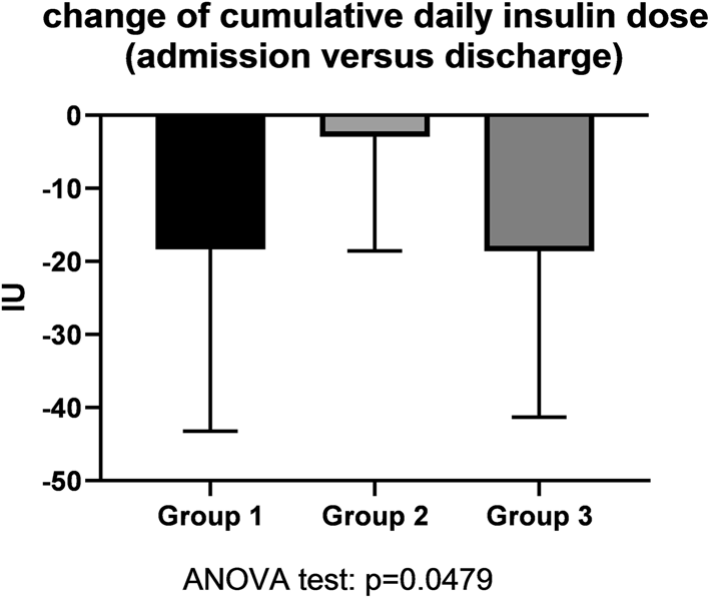
Change of cumulative daily insulin units (IU) from the time of admission versus discharge. Asterisks represent p values in post-hoc analysis

The number of hypoglycemic episodes per 24 h of CGM was highest in the positive-control Group 3. Group-1 and Group-3 patients did not differ with regard to hypoglycemic episodes per 24 h though (Fig. 4). When comparing the average number of nocturnal hypoglycemic episodes per 24 h of CGM, the same proportion of hypoglycemic episodes was found (Fig. 5) and patients of Group 1 and Group 3 did not differ. In other words, diabetes patients both with a hypertensive crisis (Group 1) and with symptomatic hypoglycemia at admission (Group 3) had a high number of hypoglycemic episodes during hospitalization. By discharge, the daily cumulative insulin dose decreased to the same extent in Group-1 and Group-3 patients (Fig. 6). Of note, insulin therapy was abandoned completely in 3 or 15.8% of Group-1 patients, in 2 or 10.5% of Group-2 patients and in 2 or 13.3% of Group-3 patients by discharge.

### Plasma norepinephrine, a surrogate of sympathetic tone, was elevated in hypertensive diabetes patients

**Fig. 7:**
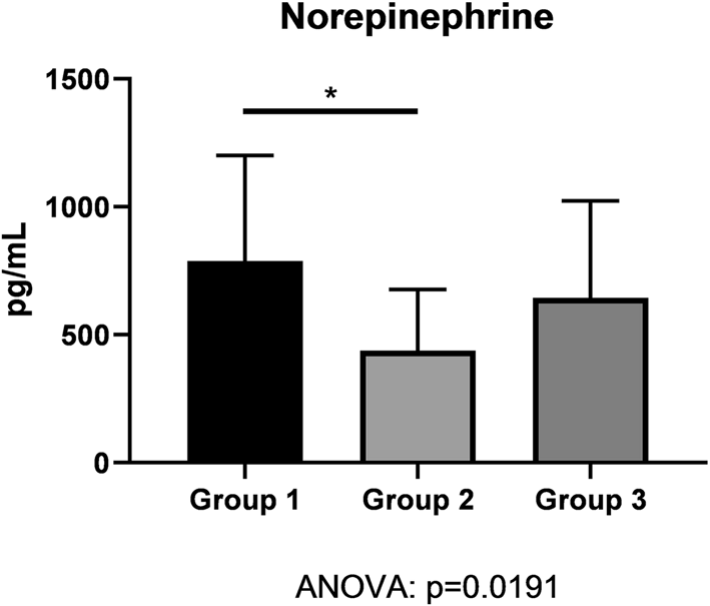
Plasma Norepinephrine levels at admission among groups. Asterisks represent p values in post-hoc analysis

Plasma norepinephrine was higher in Group-1 patients when compared to control patients of Group 2 (Fig. 7). There was no difference in plasma norepinephrine concentration between Group-1 and Group-3 patients. Heart-rate variability was not compromised among all patient groups (Table 1). Moreso, relevant differences in the HRV across the groups were not found.

### Mean systolic arterial blood pressure at daytime was not different in diabetes patients with hypertensive crisis compared to the ones with hypoglycemia on admission

**Fig. 8:**
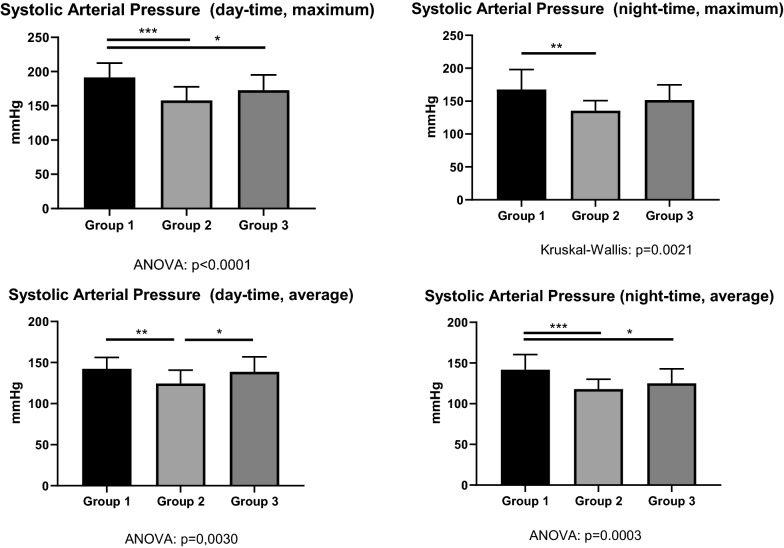
Maximum systolic blood pressure both at daytime and at nighttime (upper panel) and average systolic arterial blood pressure both at daytime and at nighttime (lower panel). Asterisks represent p values in post-hoc analysis

Maximum systolic blood pressure was highest in Group-1 patients both at daytime and at nighttime (Fig. 8, upper panel). Conversely, the average systolic arterial blood pressure was not different between Group 1 and Group 3 both at daytime and at nighttime (Fig. 8, lower panel). By discharge, the use of antihypertensive medication was intensified in Group 1. However, there was no relevant change in both the number of antihypertensive classes and the defined daily doses (DDD) of the antihypertensives by discharge, in Group 2 and Group 3, when compared to admission (Table 2). Within 24 h before discharge, the average mean arterial pressure was well controlled in all study patients: Group 1: 95.3 mmHg, Group 2: 89.3 mmHg, Group 3: 97.1 mmHg (p = 0.12).

### Renal function was impaired in diabetics with hypertensive crisis at admission

By discharge, group-wise changes of serum creatinine were not different among groups (Table 2). During hospitalization, 1 out of 19 Group-1 patients, 3 out of 19 Group-2 patients, and 1 out of 15 (38.5%) Group-3 patients had an evolving AKI. After exclusion of serum creatinine of patients with an evolving AKI in hospital, eGFR at discharge was less in Group-1 patients than in Group-2 patients (Fig. 3). However, Group-1 and Group-3 patients were not different in terms of eGFR at discharge.

## Discussion

Beyond hypoglycemia, insulin exerts sympathoactivatory [12] and parasympathoinhibitory actions [13]. Insulin’s anabolic action may contribute to weight gain, which independently associates with sympathoactivation and arterial hypertension [14]. In the long run, hyperinsulinemia without overt hypoglycemia may contribute to arterial hypertension. In contrast to intrinsic sympathoactivating properties of insulin [12], acute sympathoactivation leading to norepinephrine release in pivotal brain areas occurs following severe hypoglycemia [15]. Importantly, diabetic autonomic neuropathy as part of diabetic neuropathies [16] and hypoglycemia unawareness represent serious complications of diabetes mellitus that may affect the hypoglycemia responsiveness of the autonomic nervous system. Although these entities were not exclusion criteria in this study, a prevalent cardiac autonomic neuropathy among study participants is unlikely because 24-h HRV was not reduced [17], and resting heart rate was not increased [18]. Nevertheless, future studies on hypoglycemia counterregulations need to comprehensively assess the existence and, if applicable, the manifestations of both diabetic autonomic neuropathy and hypoglycemia unawareness. In analogy to a previously published clinical study [9], the current study aimed to detect a possible association between glycopenia and hypertension. As a selection criterion, the maximum systolic arterial pressure was highest in Group-1 patients. Likewise, hypoglycemic episodes per 24 h CGM were more often detected in the overtly hypoglycemic Group-3 patients when compared to control patients of Group 2. Of note, the first symptomatic hypoglycemia occurring at admission was the inclusion criterion for Group-3 patients and did not count as a result. As the main finding, the high number of hypoglycemic episodes in hypertensive Group-1 patients and the elevated mean arterial blood pressure detected in initially hypoglycemic Group-3 patients were unexpected.

### Evidence for a higher-than-optimal daily cumulative insulin dose in diabetes patients both with hypoglycemia and hypertensive crisis at admission

As the primary outcome, the number of hypoglycemic episodes per 24 h of CGM, suggests, this pilot study focused on the hypoglycemic burden in insulin-treated diabetes patients of any causality. In Group-3 patients, the hypoglycemic burden is evident by the proven hypoglycemic episodes during CGM, and by the reduced daily cumulative insulin dose by discharge. Therefore, we conclude that Group-3 patients had a higher-than-optimal daily cumulative insulin dose at admission. Surprisingly, hypertensive Group-1 patients and initially hypoglycemic Group-3 patients showed no difference with respect to hypoglycemic burden in the CGM results. Likewise, as evidence for a higher-than-optimal insulin dosage, the daily cumulative insulin dose was decreased in Group-1 patients by discharge.

### Hypoglycemia-induced neurohumoral stimulation may represent one cause for hypertensive crisis in insulin-treated diabetes patients

Group 1 and Group 3 had a similar number of hypoglycemic episodes per 24 h CGM and similar norepinephrine plasma levels. In addition, both systolic and diastolic blood pressure were higher in patients of Group 1 and Group 2 than in control patients of Group 2. However, both day-time maximum, and night-time average systolic arterial pressure were higher in Group 1 in comparison to Group 3 (Fig. 8). The higher blood pressure of both Group-1 and Group-3 patients when compared to Group-2 controls may be due to sympathoadrenal activation following repetitive hypoglycemic events, as the reduction of insulin dosage during hospitalization suggests (Fig. 6). Hypoglycemic events may have triggered the norepinephrine release and the blood-pressure increase in Groups 1 and 3 within the framework of the Somogyi effect. The difference between Group 1 and Group 3 likely relates to the body-mass index being higher in Group-1 patients (Fig. 2). A propensity for arterial hypertension in the framework of metabolic syndrome is likely. Hypothetically, leptin—mediated activation of sympathetic nerve traffic may have contributed to the higher systolic blood pressure in Group 1 in comparison to Group 3 [19]. Long-term studies are needed to address the question, whether fear of or repeated hypoglycemic events lead to higher sugar intake as a contributing factor to obesity [20]. Besides obesity, a more advanced chronic kidney disease found in Group-1 patients may further contribute to the evolution of arterial hypertension either via a renoparenchymal or renovascular mechanism [21]. Either way, the resulting poorer arterial hypertension may lead to vascular calcification and a faster decline of kidney function [22]. Clearly, Group-1 patients had more than one single mechanism contributing to the hypertensive crisis at admission.

### Repetitive hypoglycemia as a cause for prolonged sympathoactivation?

**Fig. 9:**
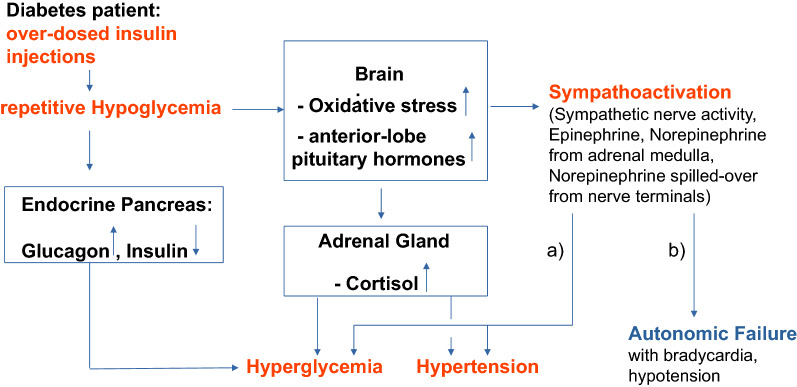
Flow chart of events that link repetitive hypoglycemia due to over-dosed insulin with hypertension. **a** Resources of neurotransmitters and hormone precursors are not exhausted, **b** resources of neurotransmitters and hormone precursors are exhausted. *< 0.05, **< 0.01, ***< 0.001, ****< 0.0001

Tsujimoto et al. [23] analyzed the outcome of 414 diabetes patients presenting with severe hypoglycemia (< 50 mg/dL). The initially elevated blood pressure dropped back to normal within 2 h after the start of hypoglycemia treatment. From these data, the activation of both plasma catecholamines and sympathetic nervous system may take up to 2 h, which likely depends on the severity and the duration of the hypoglycemic episode. As for hypoglycemia, the concept of “hypoglycemia-associated autonomous failure” with a combination of hypoglycemia unawareness and attenuated responses to repetitive hypoglycemia has been established [24]. One explanation of autonomous failure may be the exhaustion of hormones on the levels of the anterior lobe of the pituitary gland which is activated by hypoglycemia, and on the level of presynaptic stores of neurotransmitters of the sympathetic nervous system due to repetitive hypoglycemia. Hypothetically, in analogy to obstructive sleep apnea (25; 26), repetitive hypoglycemia may lead to a neurohormonal stimulation including a prolonged sympathoactivation (Fig. 9). Based on levels of reduced glutathione in the brain of an animal models, brain areas such as the hippocampus, striatum and parietal cortex need up to 24 h to replenish reduced glutathione after a series of moderate hypoglycemic episodes. Repetitive hypoglycemia may exacerbate oxidative stress in the brain [27] with possible implications for central-nervous-system regulation of sympathetic tone [28].

### Limitations

The study size of this pilot study was small. CGM and ABPM results were not blinded and could have influenced therapy decisions. From the literature, the yielded hypoglycemic rate is likely to be decreased by the open-label flash CGM used in all patient groups of this study [29]. Therefore, the high rate of hypoglycemic episodes in Group 1 is unexpected. Although flash CGM technology was proven to be accurate in comparison to capillary-blood glucose tests [30], technical limitations include a period of 12 h after placing the senso when flash CGM readings are unreliable [31], inaccurate CGM values during rapid excursions of blood glucose [32] and during exercise [33]. Overall, the accuracy of flash CGM was shown to be slightly lower in the hypoglycemic range, though more hypoglycemic episodes were detected [34]. When reporting daily cumulative insulin dose, within-day dosing issues were not considered. To assess the autonomic nervous system comprehensively, non-invasive techniques such as power-spectral analysis of heart rate would be needed in addition to HRV. To gain a comprehensive picture of the hypoglycemia—hypertension relationship, a better coverage of blood-pressure and tissue glucose monitoring is needed. As the use of long-term CGM becomes feasible, future studies may even provide insights on the initial hypoglycemic episode leading to hospitalization.

## Conclusions

In this study, we demonstrated an association between hypoglycemic episodes, norepinephrine release and uncontrolled arterial hypertension in diabetes patients on insulin therapy. The results of this pilot study may encourage health-care professionals to put hypoglycemia on the list of causes to consider in hypertensive diabetes patients. In addition, given the fact that Germany is a country with a high use of insulin per capita [35], the results of this study may increase the awareness to minimize the incidence of hypoglycemic episodes in insulin-treated diabetes patients.

## Data Availability

All relevant data are provided in the paper. Source data are available on request from the authors.

## Abbreviations

ABPM: Ambulatory blood-pressure monitoring
AKI: Acute kidney injury
DDD: Defined daily dose
eGFR: Estimated glomerular filtration rate
CGM: Continuous glucose monitoring
HbA1c: Glycated hemoglobin A1c
IU: Insulin units including units of insulin analogues
n: Number
